# Observations on Injuries and Diseases of the Rectum

**Published:** 1833-10-01

**Authors:** 


### Medico-Chirurgical Review,

### No. XXXVIII.

JULY 1 TO OCTOBER 1, 1833.

### I.

Observations on Injuries and Diseases of the Rectum. By *Herbert Mayo*, Esq. F.R.S. Surgeon of the Middlesex Hospital. Octavo, pp. 220. Burgess and Hill, London, 1833.

WE need not introduce Mr. Mayo to our readers; he is sufficiently and favourably known to them already. Neither need we say much on the subject which he has selected. We believe that the profession are generally of opinion, that it is one very capable of further elucidation than it has received, and that much uncertainty, if not obscurity, still hangs about it. When we add to this consideration, the circumstance of many insulated facts and valuable papers on particular affections of the rectum having been published within these last few years, we may fairly admit that a work which shall embody all that is now known, and contain, in addition, some novel reflections or suggestions, some original practical hints, and some new modes of viewing established facts, is not undeserving of attention. We think no further preface necessary, but shall proceed at once to the examination of Mr. Mayo's work.

It is divided into eight chapters, treating severally of fissure and laceration of the rectum—of protrusion of the bowel—of hæmorrhage and pain—of piles—of fistula—of constipation and the use of instruments—of stricture of the rectum—and, finally, of cancer of the rectum. On some of these subjects, little, probably, can be said that is new; but, in others, there is much scope for original remark, and for useful improvements in treatment.

### I. Of Laceration of the Rectum.

This chapter embraces not only laceration of the rectum, but ulceration of the gut also, affections not necessarily connected with each other. Mr. M. observes that it is surprising that laceration is not more frequent, in consequence of the thinness of the parietes. Mr. M. relates two cases.

*Case* 1. A lady, after her confinement, had great irregularity of the bowels, which seldom acted without medicine, and then with much pain. On one of these occasions something appeared to give way, and a sense of exquisite soreness at one part of the rectum was superadded to her former sufferings. On examining the bowel, Mr. Mayo found a small transverse fissure in the lining membrane, at the back part of the bowel, immediately within the sphincter. There was a little hardness round it, and it was acutely sensible. The bowel above was large and relaxed, and contained at the time a considerable volume of fæcal matter.

"I recommended that the bowel should be immediately washed out with warm water, by which means its contents were brought away: that every morning the injection of warm water should be repeated, with the object of completely unloading and relieving the rectum daily: and that at night a mild mercurial ointment should be applied to the fissure of the mucous membrane. By steadily pursuing this plan, the lady in a short time recovered. Occasionally, since her recovery, when the action of the bowels has become irregular, she has been threatened with a return of the complaint; but, upon resuming the use of the remedies by which the part was before restored, she has in each instance found the uneasy sensations cease." 4.

In the second case there was a fissure, but no history, so far as we can see, of laceration. The patient, a gentleman, got well by taking every night a grain of blue-pill, with three of compound extract of colocynth, and applying the mercurial ointment as in the former case. Mr. Mayo's principle of treatment, in these cases, is to keep the bowels properly open, and to apply mercurial ointment. We suppose that, if the former requisite be attained, any moderately-stimulating application would answer as well as the mercurial ointment.

*Ulcer of the Rectum.*

This may or may not originate in laceration. When neglected, the complaint is serious and difficult to manage. There is great pain; mucus, purulent matter, and occasionally blood are discharged with the fæces, and some extent of ulcerated surface is felt upon examination. We would observe that the ulcer is usually at the posterior part of the gut, opposite the os coccygis, that it is frequently small, and circular, that it is recognized by the extreme pain produced by the finger of the examiner touching it, and that usually a little blood follows the examination.

Mr. Mayo recommends the remedies directed for the preceding affection —leeches to the mucous membrane at the anus, if the pain be severe, and suppositories of opium or belladonna—the application of lunar caustic to the ulcer. If these means fail, Mr. Mayo advises that the sphincter and the ulcer be divided by a vertical incision with a bistoury or a scalpel, the edges of the incision being temporarily kept apart by lint introduced into the wound. As this plan of treatment must be familiar to most of our readers, we need not stop to notice a case brought forward by Mr. Mayo. In the Number of this Journal for January, 1831, will be found the substance of some observations on ulcer of the rectum, by Mr. Colles, in which that able surgeon describes the affection with great accuracy, and gives ample directions for the division of the ulcer.*

We believe, however, that simple division is not always successful. Mr. Brodie, investigating this subject with his usual patience and acuteness, has come to the following conclusions on this subject. Division of the ulcer and

* See Journ. cited, pp. 223 and 224.

sphincter is useful by the rest which is thus given to the former. But the sphincter muscle is not exactly circular, but arises from or is virtually attached to the os coccygis behind, whence its fibres proceed on either side of the extremity of the gut to the tendinous line in front, where it meets the perinæal muscles. Now the fixed points being behind and before, a vertical incision in the median line posteriorly, may not divide the muscular fibres, or, at all events may not so divide them as to prevent their action. The ulcer of the rectum is usually in the centre, over the os coccygis. Hence Mr. Brodie makes two incisions, one through the ulcer and one upon the side of it. By these the effectual division of the muscle is ensured, and, at the same time, the benefit of a division of the ulcer itself, if there be any, is obtained.

It is well to know, that ulcer of the rectum may occasionally become a cause of death. In a patient whom we saw, a gentle examination appeared to bring on enteritis, the result of which was fatal.

*Complete Laceration of the Rectum.*

Mr. M. relates a case of this kind.

"————, ætat. 40, naturally of a very constipated habit of body, and at the time being on a journey, on striving to relieve the bowels, which had not acted for many hours, felt something give way, to use her own expression, and on the following morning some fæces passed *per vaginam*. On examination by the vagina and rectum, a transverse rent was found two inches within the parts, sufficiently large to admit the end of the finger. The only treatment adopted in this case consisted in frequently and carefully cleansing the part by injections of water, and regulating the state of the bowels by proper medicines. The patient entirely recovered. In five weeks the fæces had ceased to pass per vaginam." 13.

If the opening shews no disposition to contract, the nitrate of silver, or some other escharotic may be employed, in combination with an elastic gum pessary. We see nothing deserving of attention in Mr. Mayo's observations on recto-vaginal fistula. He says nothing of the application of the actual cautery in these cases. Many think highly of it.

Mr. Mayo relates a case, of which we have seen several examples. A young man presented himself with dark diffuse inflammation, tending to sloughing of the perinæum, scrotum, and penis. Six weeks previously, after costive bowels, he had a looseness, and a lump seemed to him to have formed in the perinæum. In two days afterwards the swelling of the scrotum and penis had begun. A deep incision was made, we presume by Mr. M., in the perinæum, and the fluid which flowed had a fæcal odour. In a few days fæcal matter began to escape through the wound, establishing the certainty of a communication between the perineal cellular tissue and rectum. Mr. Mayo seems doubtful whether this was a case of laceration of the rectum, or abscess external to it in the first instance. We have seen four or five cases of this description, two, if our memory serves us, fatal. They appeared to be owing to abscess external to the gut, penetrating the latter, and giving rise to sloughing of the cellular membrane consecutively. It is not uncommon to find a fish-bone or some foreign substance in these deep, foul, perineal abscesses. We think we have heard Mr. Brodie relate some very remarkable instances of this kind.

Mr. Mayo observes that the rectum may be torn through by violence in the introduction of instruments, as enema-tubes, within it. At Bartholomew's Hospital there is a preparation from the body of a patient whose death had been occasioned by the injection of a pint of water-gruel into the abdominal cavity through the torn rectum. We have heard of two or three instances of laceration of the rectum by a glyster-pipe. They occurred in a hospital.

Mr. Mayo observes that the most frequent lacerations of the rectum are those which occur from the pressure of the child's head in labour. These are alluded to in all works on Midwifery, and we need only advert to a method employed by Mr. Copeland in a case of complete laceration of the perinæum and sphincter. He divided the sphincter laterally, and the laceration was cured. The principle is obvious. Acting on this hint Mr. Mayo operated in a case which occurred to him soon afterwards.

*Case.* The woman had been delivered eight days previously, and the fæces passed freely through the vagina by a gaping fissure nearly an inch in length.

"As the edges of the fissure were not cicatrized, I thought the present a very favourable opportunity for repeating Mr. Copeland's operation. To give the parts every chance, I divided the sphincter muscle upon both sides, performing therefore on either side the operation for fistula ani. A small strip of lint was introduced into each wound. The edges of the original rent were afterwards washed daily with a solution of nitrate of silver, and fresh lint was replaced in the incisions as often as it was removed by the passage of fæces. The original rent healed very speedily: when it was nearly closed, the lateral wounds were allowed to unite. In five weeks from the operation the incisions had healed, and the patient had recovered the use of the sphincter. She has continued perfectly well to the present time, and was safely confined of another child in November, 1832." 25.

In cases of long-standing Mr. Mayo intends to employ this method in combination with paring of the edges, ligatures, &c.

### II. OF PROTRUSION OF THE RECTUM.

This, which is commonly known by the term prolapsus, or procidentia recti, is a troublesome affection, and has excited the attention of several able surgeons. Mr. Hey, Mr. Copeland, M. Dupuytren, and more recently Mr. Fletcher, have severally written papers on the subject, each proposing some modification of the operation of excision of the ligature. We confess that we see little to arrest us in this chapter of Mr. Mayo's.

He remarks that costiveness is a frequent cause of the affection—that it is more common in children than in adults, in consequence of the greater mobility of the bowels in the former—that the remedy consists in the reduction of the gut and the insurance of regularity of the bowels by proper treatment—that in severe cases, especially adults, an operation is necessary—that prolapsus is occasionally attended with piles, or with thickening of one or more folds of the mucous membrane, and that, in such instances, the removal of the excrescence must be the first object—and that prolapsus, when slight, is most apt to be mistaken for piles. This is the gist of the chapter, and our readers may perceive that it contains little that is new.

We will notice two or three of the cases related.

1. *Prolapsus in Children.* "I attended two children in another family, the one three, the other four years of age. Both laboured under eversion and protrusion of the bowel, which took place at each motion, and required pressure to replace it. These children were of a delicate habit, but with each the bowels acted for the most part with regularity.

The method which I adopted with success in these cases, consisted in giving tone to the part by means of astringent injections. For the youngest, which was a girl, I prescribed two ounces of the infusion of catechu as an enema, to be used daily; for the elder, a boy, I ordered three ounces of the same infusion, with six grains of acetate of zinc. The remedy was administered in the morning, before the children rose; and they were kept in bed for half an hour afterwards, in order that the injection might be retained. Both the children recovered under this treatment; the protrusion of the bowel being at first lessened in quantity, and then in frequency, till the children would pass two or three days without its recurrence. Occasional doses of opening medicine were given when necessary during the four or five weeks that the plan which I have described was pursued." 38.

2. *Prolapsus—Operation performed.* A young lady, aged 20, had suffered from head-ache, &c. for several years, when Dr. Chalmers, of Croydon, suspected some affection of the rectum, and ascertained that she had prolapsus of considerable volume, which took place after every stool, and could not be replaced without difficulty. Mr. Mayo was called in. He found, after giving an enema, that the prolapsus was about the size of an orange, the coats of the bowel not at all thickened, the sphincter extremely lax, the eversion commencing about an inch within it. The operation performed was the following.

A small fold of intestine was pinched up with forceps, and tied with a silk ligature; care was taken to include the mucous and submucous coats alone in the ligature; the whole surface included was less than that of a sixpence. Before finally tightening the ligature, the surface of the little fold was cut with scissors. Three such folds were tied upon opposite aspects of the bowel, and at different distances from the sphincter. The patient hardly felt the operation, so small is the sensibility of the internal parts of the body, unless when inflamed. The parts were then replaced. During the four days which followed the operation, the patient was not allowed to sit up; and the bowels, which had been well unloaded before, were kept confined, very light and moderate liquid nourishment alone being allowed, and an enema of laudanum having been administered. On the fourth day slight prolapsus recurred, but was replaced with less difficulty than before. On the sixth day the parts were examined. The mucous membrane, when the bowel was extruded, appeared fuller and more loaded with blood than before the operation. The little portions of membrane which had been tied had come away; but the ligatures had not yet separated, but remained fixed in the shallow ulcers which they had produced: they were removed. From this time improvement made progress daily, and in a fortnight the prolapsus had disappeared.

3. *Protrusion with Inflammation, &c.* E. R. æt. 65, had been subject for sixteen years to occasional prolapsus, which he could always reduce without difficulty. He was admitted into the Middlesex Hospital, the gut having been down for eighteen hours. It was of the size of a large walnut, swollen, of a scarlet colour, painful and extremely tender, so that he could not bear it to be pressed. The practice therefore pursued was the following. Leeches were applied to the mucous membrane of the bowel, and afterwards an anodyne poultice; and a dose of opium and calomel was administered. The following morning the tenderness of the bowel was greatly diminished, and it was returned without difficulty.

Mr. Mayo, in this notice of prolapsus, says nothing of two means of treatment which appear to us extremely important; we allude to the horizontal posture, and the Ward's paste. We have seen very lately a case of prolapsus, much more extensive than that of the young lady on whom he operated, remedied by these measures. We have one word to say on the exhibition of the paste; it should not be given when the bowels are constipated, or when there is much irritation, or any pyrexia. But after the bowels have been got into a healthy state, and irritation has been removed, we have certainly seen much benefit derived from this excellent medicine. We would refer our readers for further remarks on the surgical treatment of prolapsus to the memoirs of M. Dupuytren, and of Mr. Fletcher. The latter will be found in the number of this Journal for October, 1831.

### III. OF BLEEDING FROM AND PAIN IN THE RECTUM.

The former, Mr. Mayo avers, occurs, as well as the latter, as a substantive disease. We think that with respect to the former Mr. Mayo is somewhat illogical, for, immediately after this declaration, he makes it depend on congestion of the venous system of the abdomen, or obstruction in the circulation through the liver. If it depends on congestion only, it is obvious that it is not the disease but the symptom. If, on the other hand, it depend on enlargement of the hæmorrhoidal veins, it is still merely the symptom, and the latter is the disease. It appears very evident to us that bleeding can, under no circumstances, be considered a substantive disease. There must be a cause for it, and that cause is the disease. We make these remarks, because it is always better to discard these erections of symptoms into diseases. It is astonishing what error the ancient disposition to give names to symptoms has caused and has perpetuated. Asthma is a case in point.

"An ordinary attack of bleeding from the rectum has the following course. There is a sense of weight, heat, fulness, and general uneasiness in the bowel: this goes on increasing for twenty-four hours: then the patient observes that when the bowels act, part of the discharge is liquid; it consists of blood, which seems poured out at the time only that the bowels act; or the passage of the fæces seems necessary to rupture the small vessels from which the hemorrhage proceeds. In another day the uneasy sensations lessen, and they quickly cease altogether." 49.

Such hæmorrhage from the rectum Mr. Mayo considers a relief to the system, and a warning that something is wrong. Mr. M. recommends gentle aperients and cold bathing during the attack, and remedying what is wrong, to prevent its recurrence. But, says Mr. Mayo, when discharge of blood continues very long, it weakens the system, and then a different plan must be adopted. To illustrate this, he relates the following case, which we will give entire.

"James Tucker, ætat. 24, was admitted into the Middlesex Hospital, Oct. 6, 1829. During the three preceding months he had habitually passed blood by stool: at first he was considerably reduced in strength by this discharge, but afterwards it affected him less. The quantity of blood lost was greater at first than afterwards: at the period of his admission it amounted to two or three table-spoonfuls, which came away immediately after each evacuation. The only pain complained of was occasional numbness and aching down the inside of the thighs. This patient was directed to use an astringent enema containing ten minims of laudanum after each discharge of blood.

The effect of the injection was to constipate the bowels, and to produce a dull pain at the sacrum. The astringency of the injection was therefore lowered, and the laudanum omitted, and a few grains of blue-pill and extract of rhubarb were ordered to be taken every night. The discharge of blood gradually lessened; and on the 26th the patient left the hospital cured." 52.

In this case there is one great omission. No mention is made of the state of the rectum. It is only half a fact. But the whole subject appears to us to stand on a false bottom. Bleeding is trifling in its consequences or severe, according to the cause that produces it. We know a gentleman who is affected with hæmorrhage in precisely the manner Mr. Mayo describes. But that gentleman has enlargement of the hæmorrhoidal veins, and whatever tends to act injuriously on them tends to produce the bleeding. Again, a patient will not have long-continued bleeding without an adequate cause. What is that cause? That can only be ascertained by inquiry and examination. It may be ulceration of the colon or rectum, or internal hæmorrhoids, or some other affection. As examination alone can decide this point, so, without such examination, all treatment must be empirical—a shot in the dark. The erection of the symptom bleeding into a disease tends to such empiricism. We would recommend practitioners to be always sceptical in this admission of symptoms—to believe there is always a local cause for them—and to endeavour to detect that cause. How often have surgeons been deluded by the term typhus, and stood by while their patients were dying of unlooked-for and consequently unfound putrid abscesses.

*Pain in the Rectum.* Of this, unattended with discoverable local disease, Mr. Mayo has seen but two examples. These we will extract.

"A gentleman, about 40 years of age, sent for me during a paroxysm of pain in the rectum, but it had subsided before I saw him. He told me that two or three times a year he was liable to this seizure, which was not, that he had observed, connected with the state of his bowels, or with his habits of living. The pain which he used to experience was intense, and would last half an hour. He was not, that he knew of, liable to lose blood by stool, nor had he ever suffered from piles. Upon examining the rectum, I could discover no disease in it. The pain did not appear to arise from spasm of the sphincter.

I attended a patient with Mr. Stevenson, of the Edgeware Road, who suffered from pain in the rectum. Something less than two years before this, he had a syphilitic ulcer upon the penis, for which he had taken an unusually large quantity of mercury, owing to the difficulty of producing sensible mercurial action in his system. The ulcer however healed; but while he was recovering, and his system was yet charged with mercury, he began to experience aching pains in the incisor teeth and in the rectum. The sense of aching in the teeth and in the rectum was not constant, but would come on frequently during the day without any assignable cause. It had lasted a year and a half, during which he had remained perfectly free from symptoms of lues. This patient, who was otherwise in good hea th, suffered his mind to be greatly distressed by the continuance of the neuralgia. He was anxious to try every plan which held out the least promise of benefiting him. But of all the remedies which he tried he appeared to experience relief from one only, which was a course of sarsaparilla." 57.

The fourth chapter, occupying forty-two pages, is devoted to the consideration of piles. There is little novelty in Mr. Mayo's remarks on this affection. For internal piles he prefers the ligature to excision; he operates in the usual manner. The following case of external piles may be related.

"A physician whom I had attended for inward piles came from the country to consult me for such an attack as that which I have described. He had used the remedies which have been recommended, having applied leeches several times, and having been cupped upon the sacrum, and each time with relief; but there remained a tumour of the size of a chesnut on one side of the bowel, which was still painful on pressure, and he was in hopes that an operation would relieve him. Before he saw me, after he arrived in London, he met, and the part was examined by, a surgeon of considerable experience, who told him that he could if he please return the tumour within the sphincter, but that the pressure necessary would give considerable pain.

The appearance which the part presented was that of a solid tumour on one side of the anus extremely firm, partly covered with tense and shining integument, partly with the mucous membrane of the margin of the bowel. On examining the rectum, the swelling and hardness were found to extend an inch within it. It was evident that no operation would be of service; and that as the tenderness and pain in the part, though still considerable, were progressively lessening, no treatment would be necessary beyond the use of a poultice, and occasional doses of opening medicine, with abstinence from wine and heating food. The tumour I concluded to be an outward pile, no part of which would on its diminution be drawn or forced within the sphincter. The result proved that this opinion was right: the tumour only shrunk." 87.

When an external hæmorrhoid is very painful, elastic, tense, and throbbing, Mr. Mayo punctures it. Under other circumstances, fomentations and leeches, if necessary, are sufficient during the paroxysms of exacerbation to which both internal and external piles are subject. External piles should be removed by excision, if removed at all.

Mr. Mayo describes, as a variety of external piles, what, if we understand his description rightly, do not appear to be piles at all.

"The cases I mean are of the following description. Some soreness is felt of the skin about the anus, which gradually increases. When the part is examined, two or three red circular elevations of the skin are seen, from three to four lines in diameter. They are attended with heat and soreness. If neglected they sometimes ulcerate. They commonly get well under the use of mercurial applications, joined if necessary with mercury, administered internally. I do not know whether this affection which I have seen several times, and with one exception in women only, is of a venereal origin. I have not seen it in connexion with other symptoms of lues. But in one instance, in a woman forty years of age, it existed at the same time near the anus, and in the axilla. The complaint had begun in the first situation, and I have no doubt it had been transferred to the axilla by contact.

I saw the same affection in a child two years of age, a boy. In this case, mercurial applications irritated the part, which got well under the use of the zinc ointment. In this case there were originally two blotches, one of which became ulcerated, and the skin beside it grew into a pendulous slip, distinctly caused by the irritation of the neighbouring ulcer. As the original complaint got well, the slip of skin shrunk again and disappeared.

When these little folds of skin originate from a local cause of irritation, they generally go away spontaneously: sometimes they shrink and disappear; at other times they perish by ulceration. The most common causes of their production are gonorrhœa or leucorrhœa, when insufficient attention is paid to cleanliness." 98.

Now these productions of cutis are of every day's occurrence, and may be seen at any time in Lock Hospitals. They are usually known in these establishments by the name of condylomatous elevations and condylomatous sores, although they do not answer exactly to ordinary descriptions of condyloma. They are totally distinct from hæmorrhoids, for the latter originate in congested veins, whereas these are always vascular productions of the cutis, highly organized, and the result of cutaneous irritation. We have under our observation, at the present moment, at least half a dozen of these cases, and we feel surprised at Mr. Mayo's having seen so little of the affection.

We have said that they are the result of cutaneous irritation, and any discharge, with want of cleanliness, is sufficient to produce them. They are frequently the result of gonorrhœa or of leucorrhœa, as Mr. Mayo has remarked, but they are also, in many instances, a distinct syphilitic primary symptom, capable of communication, and followed by secondary symptoms, usually enlarged tonsils, with superficial ulceration, and the syphilitic psoriasis, lepra, tubercle, or stain.

When syphilitic, these productions require mercury.

The fifth chapter is on fistula ani. We do not believe that much of novelty can be said on such a subject. We need only allude to one or two insulated passages. Mr. Mayo gives the following symptomatology of deep-seated abscess near the rectum.

"It is to be suspected, when the patient experiences aching and throbbing pain in the part, often not constant, but recurring at intervals, and frequently with a spasmodic character, the pain being aggravated on the passage of the fæces, and the complaint attended with symptomatic fever. The abscess often does not declare itself by any external fulness or prominence; and its existence can only be ascertained through an examination of the rectum; when, at some part which is more tender than the rest of the mucous surface, a fulness and fluctuation, if the abscess is matured, are felt." 104.

Mr. M. recommends, as all have done, an early opening of these deep-seated abscesses. Indeed, if there is reason to believe that matter is forming by the rectum, Mr. M. advises that the inflamed part should be punctured deeply with a lancet, even if no fluctuation can be felt. In illustration of the relief experienced from this practice, Mr. Mayo relates a case.

The patient was a young lady, and there was general fulness of the nates on one side, towards the anus. The lancet was introduced to some depth, but blood only followed; it was passed still deeper, and a little matter escaped. The patient was relieved, and soon recovered.

Mr. Mayo relates a case of abscess within, or close to the prostate, opening into the bowel.

"Wm. Knight, ætat. 65, was admitted into the Middlesex Hospital, August

9, 1832. For five months previously, he had experienced violent aching pains about the hips and loins, and down the back of the thighs to the knees, slight dysuria, and habitual constipation of the bowels. During the last six weeks he had suffered more acute pain within the anus, shooting to the projections of the ischia and round the haunch bones. He passed urine with great difficulty, and could scarcely void it unless at the same time he strove to empty the bowels. Upon examining the rectum, I found a collection of fluid in the region of the prostate gland. This patient experienced relief from the use of the hip bath, with an opiate suppository at night, and mild aperient medicines. But in five days after his admission, the abscess broke into the rectum, discharging as he thought a pint of matter, which was followed by the complete removal of all his symptoms, and a very speedy recovery. He left the Hospital perfectly well on the 28th of September." 111.

For an abscess to do well, matter must escape from its cavity freely. Mr. Mayo remarks that this is not to be effected so much by a large opening as by several independent situations. This, however, must be entirely determined by the circumstances of the individual case.

Mr. Mayo denies that it is the action of the sphincter that prevents the cure of the fistula ani. He maintains that this sinus shews no more indisposition to heal than any other sinus in cellular membrane. But, as Mr. Mayo allows that the action of the sphincter is very operative in preventing the healing of lacerations of the rectum, and of ulcers of the gut, it appears to us that he cannot, with any grace, deny its action in fistula. If it acts injuriously in one instance, there is no good reason why it should not act in another.

If fistula ani be complicated with urinary fistula, the patient, observes Mr. M. may be cured, if he has a good constitution. The plan recommended is to close the urinary fistula first, "which may often be done by regulated diet and medicine alone, unless there is stricture of the urinary canal." When this is closed, then the fistula ani may be cured by the common operation.

The sixth chapter treats of constipation of the lower bowels, and of the use of instruments.

Mr. Mayo observes that constipation may depend on one or other of these three causes. Either the secretions are wanting, of which, combined with the refuse of the aliment, fæces are formed; or, fæces being formed, there is not liquid secretion enough for their expulsion; or, the fæces being of a proper quantity and consistence, either they are not of a quality to stimulate the bowels to action, or the muscular fibres of the bowel being enfeebled have not force enough to expel them.

Mr. Mayo relates a case of what he styles want of fæces, and of the consequences which result when the blood is not relieved from this excretion. We will give it entire.

"A young gentleman, ætat. 25, consulted me, labouring under the following symptoms. He complained of being oppressed with languor, and described himself as incapable of any effort mental or bodily. Frequently during the day he was drowsy and disposed to sleep, and at night he slept long and heavily. He considered that these symptoms, under which with certain intermissions and with variations in their degree he had laboured for several years, depended upon constipation of the bowels. If it happened that the bowels were well relieved in the morning, the oppression which he suffered seemed for that day lightened of half its weight. In the preceding autumn he had been in the country taking considerable exercise daily. At that time the action of the bowels had been regular, and he had felt himself perfectly well.

For two months before I saw this patient, he had been endeavouring, by means of medicine, to make up for the want of bodily exercise. He had used injections, but they seldom brought away fæces: he had taken various medicines, but they had generally produced watery motions, which used to lower instead of relieving him. His tongue was clean, his appetite good. There was no embarrassment in the early stages of digestion; no sense of weight or uneasiness at the stomach; no acidity, distension, or flatulence, after his meals. The only bodily sensation which he complained of, was a sense of uneasiness about the middle of the belly. This uneasiness was greatest, when the bowels were most confined: at such times he could not draw himself fully upright without pain about the umbilicus, which was increased by pressure.

This patient recovered his health upon taking a course of medicine, which produced daily a full action of the bowels. The medicine which most contributed to this purpose consisted of equal parts of scammony, gamboge, aloes, and the compound extract of colocynth." 127.

We confess that we are not convinced of there having been a want of fæces in this case. What was wanted was the power of getting rid of them. This gentleman took little or no exercise, and had a good appetite. What did he with his food? The excrementitious part must have become excrement—it was not voided—where was it? The fact is, that this gentleman's colon was loaded, and all that was necessary was to empty it, and prevent its being loaded again.

Mr. Mayo criticises, and we think very fairly, Dr. O'Beirne's statement, that fæces are seldom found in the rectum. Whoever has had occasion to examine the gut often, must have found upon his finger very unequivocal marks to the contrary.

Mr. M. remarks that constipation may arise either from the fæces not stimulating the bowel, or from the muscular coat of the latter being weakened or palsied. Deficient biliary secretion will produce the former.

Accumulation of fæces in the great intestine, in elderly women, often has its seat in the rectum, and relief is obtained from emptying the bowel mechanically. Mr. M. relates a case of this sort. If the accumulation be higher, a long flexible tube, as recommended by Dr. O'Beirne, may be serviceable. If the accumulation takes place beyond the reach of instruments, we must resort to drastic purgatives.

Sometimes constipation seems attributable to affections of the spine. Mr. Mayo relates an instance of this in a young lady. The lumbar portion of the spinal chord was softened for the length of two inches.

In the treatment of constipation, Mr. M. recommends punctuality in attendance at the water-closet—regular exercise—aperient medicine, if necessary, and the use of the bougie, injecting syringe, or lavement. Mr. Mayo is not very precise or very full in his remarks on these modes of treatment.

The two last chapters are on stricture of the rectum and cancer of the rectum.

### Stricture of the Rectum.

Mr. Mayo thinks that no one part of the rectum is more disposed to spasmodic stricture than another. The cases he has seen have left him with the impression that the upper part of the rectum and the sigmoid flexure of the colon, are liable to irregular contractions of their muscular tunic, capable of obstructing the passage of the fæces and of making resistance to the introduction of instruments. This irregular action is generally dependent upon a vitiated state of the secretions; and is more frequently relieved by a regulated diet and alterative medicines, and the use of injections, than by the employment of instruments. Nevertheless the use of the bougie is sometimes beneficial in spasmodic contraction of the rectum.

Mr. Mayo relates the case of a physician who cured himself by taking nothing in the shape of food that could by possibility irritate the stomach or bowels, and leaving them to act of and for themselves, when they could no longer retain their contents. The next case is probably, nay obviously, not one of mere spasmodic contraction. As it is interesting we will give it in the gentleman's own words.

"A gentleman, now in his fifty-eighth year, who from early youth had been subject to a very irregular action of the bowels amounting frequently to an alternation of costiveness and dysentery, was about fifteen years ago strongly urged by a physician to abstain from medicine, and to let the bowels alone. This experiment was tried with great resolution. In spite of much suffering and increasing feverishness, the patient took no medicine for more than a week. Inflammation however ensued. Intolerable pain in the abdomen, and simultaneous vomiting and purging, reduced him to an alarming state in the middle of the night. Skilful medical assistance was fortunately obtained without loss of time, and the acute symptoms were subdued. The patient's general health however grew worse. His bowels were never at rest. Acrid mucus was incessantly formed, and frequently passed, leaving the sufferer in a state of great weakness. Blood was sometimes observed in the mucus. Scarcely any thing was passed without great effort and pain. Spasmodic contractions of the rectum were constantly attendant on every attempt to ease it. Great emaciation and prostration of muscular power took place, as also restlessness at night, amounting sometimes to the most painful startings from sleep. After a few ineffectual attempts to perform a cure, treating the case as one of liver derangement, he confined himself to the use of the common purgatives for the paroxysms of the complaint, and of a small quantity of rhubarb and ginger before dinner, for the daily symptoms. Though very slowly, yet he improved from year to year; but owing to the unsettled state of the bowels, he could hardly venture out of his house. By the advice of a friend, he tried, about two years ago, the daily use of lavements by means of Read's syringe. He has used nothing but tepid water. At first the lavement produced great nervous weakness; but this symptom disappeared in a short time. At present he enjoys a certain degree of comfort and ease, which entirely depends on the use of the lavement early in the morning. From a local examination it has lately been ascertained, that the rectum is contracted to about half an inch diameter, at a distance of about five inches from its termination. The daily passing of a wax bougie, softened by heat, is attended with little or no pain. The distention of the contracted part by this mechanical means relieves the spasmodic contractions, which the patient frequently feels a little above the sigmoid flexure." 163.

Mr. Crosse, of Norwich, communicated a case to our author, of what was supposed to be stricture of the rectum. Her medical attendant employed in no very gentle manner a firm bougie, and perforated the coats of the bowel at the sigmoid flexure. The patient died of peritonitis. On dissection the rectum was found to be capacious for an inch or two above the anus, but beyond this, for eight or nine inches, it was so contracted as to admit an instrument only half an inch in diameter, and its coats at the same time were very delicate and attenuated.

With respect to permanent stricture of the rectum, Mr. Mayo observes that in nineteen cases out of twenty it is seated within reach of the finger. The treatment recommended is, of course, the bougie, used with gentleness. Mr. Mayo observes that roughness may produce peritoneal inflammation, and that although the rectum at the part affected is not covered with peritoneum, yet there is so singular a consent between the pelvic mucous passages and this membrane, that it often inflames in consequence of roughness exercised upon them. We apprehend that Mr. Mayo has overstated this consent, and that the matter, if examined, will be found to stand thus. Injury done to the mucous membrane of the gut produces inflammation of that membrane, which extends by contiguity to the cellular tissue, and spreads along that tissue so as to implicate the peritoneum. This has been the case in three or four dissections which we have made. We have found the same thing occur with respect to the œsophagus. An ulcer of the œsophagus was mistaken for stricture, and a bougie was passed. It occasioned extreme pain. In a day or two afterwards the patient was attacked with typhoid symptoms and pleuro-pneumonia. She died. We examined her body, and found that the surface of the ulceration had been abraded by the bougie; that inflammation had extended from this to the contiguous cellular membrane, and had travelled along that membrane to the pleura and lung. We apprehend then that this consent between the mucous membrane of the rectum and the peritoneum, is, in the majority of cases, nothing more than the extension of inflammation.

Mr. Mayo remarks that, in some cases, the stricture may be divided with a probe-pointed knife, in addition to the use of the bougie: of course some cases are more adapted than others for this operation. Mr. M. says he would not recommend it, "except under peculiar circumstances." What those circumstances are he does not say. The narrow stricture, not easily dilatable by the bougie, is, on the whole, best adapted for this operation. There is, at the present time, in St. George's Hospital, a woman with such a stricture. It is seated about two inches from the anus, and forms little more than a narrow ring. It has been treated by bougies for six months ineffectually. In such a case division of the stricture is likely to be very serviceable.

Mr. Mayo relates a case illustrative of the difficulty sometimes experienced in distinguishing stricture from incipient carcinoma.

" A lady about forty-five years of age had suffered severely from piles, which were removed five years ago by the ligature. They did not grow from the forepart of the rectum. Some months after this the lady began to feel a tightness and sense of obstruction in the rectum. These sensations gradually became more distressing: much effort and straining were necessary to pass the fæces, which were narrow, flattened, and in fragments. After two years of suffering, this patient consulted me. There was an induration, which began two inches within the rectum, and occupied two-thirds of the circumference of the gut. The central and broadest part of the induration was towards the vagina; at this part it was two-thirds of an inch in depth. The part was acutely sensible. I recommended that the bowels should be relieved every morning by means of a lavement of tepid water, and that a soft wax bougie should be introduced into the narrowed part every second day. Under this treatment, combined with the occasional use of aperient medicine, a decided amendment took place: the narrowed part yielded to a certain extent, and there was a proportionate alleviation of all the symptoms. But in a short period the patient became worse again; the introduction of the bougie now gave more pain; it was therefore discontinued. The passage was indeed certainly freer, but the induration towards the vagina was not lessened. Under these circumstances I wished Mr. Copeland to see the case with me. The impression which the examination made upon our minds was, that the disorder was likely to prove carcinoma.

The plan which the patient followed was slightly modified. The use of the bougie for a time was not resumed. The increased sensibility of the part went away. But it was not long before the patient again complained of the contraction returning; upon which the bougie was again used, but for a shorter period than on the first occasion. Since then at intervals the patient has occasionally had recourse to this remedy again. She is now materially better: the narrowing has lost its doubtful character: the induration is less in extent, and the projecting band has little more than the character of a thickened fold of mucous membrane. Some discharge of matter *per vaginam* took place, and continued for several weeks, about a year ago. I am disposed to think that it proceeded from the induration, which may have suppurated, and the abscess have broken into the vagina at that time." 177.

Mr. Mayo mentions that ulceration may take place above the stricture, in consequence of the pressure exercised by the fæces. Mr. M. relates two cases of stricture at the sigmoid flexure, communicated to him by Mr. Cæsar Hawkins. He adverts also to an affection described by Mr. Chevalier and Mr. Earle. The rectum having been dilated by large fæcal accumulations, the upper portion of the gut is liable to become invaginated within it, and the portion thus prolapsed may become inflamed, thickened, and indurated, while the aperture through it is contracted. A careful examination is necessary to detect this state, and regulated diet, gentle aperients, the mildest injections first, and astringent injections afterwards, with the use of the bougie, are the means most likely to succeed.

The next affection is thus adverted to by Mr. Mayo.

"Spasmodic contraction of the spincter is a kind of cramp. It often comes on suddenly. The patient who has gone to bed quite well, awakes in violent pain. The spincter muscle is hard and in strong action, so that the finger cannot without great difficulty be passed into it. In some cases these paroxysms recur daily, in others only two or three times a year. In some the attack comes on gradually, and after producing uneasiness for several days gradually wears off; in others it is sudden in its invasion, and sudden in leaving the patient.

This complaint generally depends upon a confined state of the bowels; and a brisk cathartic at night, with an aperient draught in the morning, will often relieve it. In some cases the patient finds it sufficient to use a lavement of warm water, upon which the spasmodic contraction wears off.

If the pain is very severe at night, an ounce of tepid water, with twenty drops of the liquor opii sedativus, may be injected into the bowel, and at the same time purgative medicine taken. It is better to use opium in this form than as a suppository. In the latter shape, the remedy by the mechanical irritation which its presence excites has a tendency to excite the sphincter to stronger action.

Sometimes the spasm is relieved by extending the circular sphincter muscle, and keeping its fibres on the stretch. The patient for this purpose may introduce a large mould candle into the anus.

There are cases in which this disease produces long-continued and most serious suffering; in which the anus becomes permanently contracted and hardened, constituting therefore a permanent stricture, and generally combining both permanent and spasmodic contraction. The motions are passed with an effort and with pain, and all the common symptoms of stricture of the rectum are present.

In this more aggravated form, the complaint will yet often yield to simple treatment;—such as the observance of a regulated diet, the use of gentle aperient medicine, the daily use of the bougie, and of lavements of tepid water. If these milder means are insufficient, the sphincter is to be divided, and the wound, by the introduction of threads of lint, is to be made to heal by granulation from the bottom." 187.

We have not found active cathartics beneficial in spasmodic contraction of the sphincter. On the contrary, they have appeared to us to aggravate the affection. The treatment we have found succeed best was calomel and opium for a night or two, very mild aperients, as the lenitive electuary, the bidet, and, if these means failed to procure relief, the gentle employment of the bougie. We have seen many cases relieved in this manner.

### Cancer of the Rectum.

Mr. Mayo observes that there are two forms of scirrhus of the rectum.

In one the thickening is inconsiderable; but the mucous membrane is abraded, the muscular coat is hard, firm, gristly, and the canal of the bowel is narrowed. The muscular fibre of the bowel is partly converted into, partly contained in, firm, gristly, fibrous substance. This commonly begins from an inch to an inch and a half within the anus, and generally occupies from four to five inches of the bowel. Mr. M. has seen it occupy sixteen inches. The change from the healthy to the diseased state is often very gradual towards the anus, but, above, the diseased structure always terminates abruptly, meeting a raised, uneven, ulcerated edge of mucous membrane.

The second or fungoïd variety is characterised by considerable thickening, caused by the presence of a quantity of scirrhous deposit greater than in the preceding instance. The scirrhous matter, grey, fibrous, not perfectly opaque, gristly, as in the first kind, is of a looser and more succulent texture.

"Fungoid scirrhus at its commencement generally occupies a portion only of the circumference of the bowel, and is felt as a hard tumour situated about three inches within the gut and commonly upon its anterior surface, with the mucous membrane as yet unbroken. The growth of the scirrhus is liable to extend in each direction, upwards to the sigmoid flexure of the colon, and downwards so as to implicate the anus and to throw the adjacent integument into firm hard knots. Similar disease sometimes originates in and is confined to the sigmoid flexure of the colon, and is occasionally met with at parts of the great intestine still higher.

In either form of the disease, the fat external to the rectum is liable to assume a firm and crisp texture, resembling the state of the adipose membrane met with around a cancerous mamma." 196.

The patient may die gradually, worn out by suffering, or more quickly from complete obstruction of the bowels. The treatment recommended by Mr. Mayo is of the palliative kind usually adopted. Occasionally, when the canal is very much narrowed, a bougie is serviceable, as is the use of the flexible tube to wash out and unload the bowel above. Sometimes the malignant growth is so considerable, and its sensibility so great, that a bougie cannot be borne. In such cases the channel may be enlarged, if the part is within reach of the finger, by division of the scirrhus. Mr. M. relates a case of this description.

"Mary Woolgrove, ætat. 32, was recently admitted into the Middlesex Hospital. In the year 1818, she had been cut for fistula, and since that time had never been entirely free from occasional discharges of blood and mucus from the bowel. But it was not till three years and a half ago, that pain and obstruction and other symptoms of carcinoma appeared. At the period of her admission she was greatly extenuated, having suffered for several weeks constant painful purging of liquid matter. The anus was indurated, and surrounded with scirrhous nodules partly in a state of ulceration. Upon an examination of the rectum, the finger was stopped at an inch within the gut by a mass of fungoid scirrhus, through which an urethra-bougie could only be passed. By means of opiates the pain which this patient suffered was mitigated, and the purging checked ; I then tried to enlarge the passage by the use of bougies ; but the attempt was ineffectual, and violent liquid purging returned. Under these circumstances I determined to divide the scirrhus. For this purpose I introduced the blade of a strong straight probe-pointed bistoury upon the fore-finger of the left hand, and divided the scirrhus towards the sacrum, gaining space enough to allow the finger to be passed further into the bowel. I then divided in the same manner the part beyond. The scirrhus terminated, as I had anticipated, at three inches within the anus, so that the operation was entirely successful. It has given the patient great relief, who now has a free passage through the part, which is besides less sore and painful than before." 207.

Mr. M. has never seen a case of scirrhus unattended with thickening in men. We must say that we are not convinced of this form being really scirrhus. We recollect a very well marked instance of it in a female who died suddenly of inflammation of the pelvic cellular membrane. The mucous membrane was destroyed for some extent, and the other tunics were consolidated, and cut almost like cartilage. But there was ulceration in many parts of the colon, and in successive situations the disease might be traced from simple ulceration to this ulterior complication. This patient had had dysentery. We are tempted to believe that extensive ulceration of the rectum of any sort, if reduced to a chronic condition by neglect, ill-treatment, or any other cause may assume the condition described by Mr. Mayo. It is probably not a curable disease, but, if it is not actually malignant, it may be a remediable one, and unlikely to contaminate other organs.

"When carcinoma of the rectum comes before the practitioner as a hard swelling situated just within the anus, with a mucous surface as yet unbroken, the use of the bougie is not needed for the dilatation of the channel, and would be prejudicial by hastening the ulcerative stage of the disease. But other expedients suggest themselves; and we are led to inquire, whether the disease may not admit of excision, or whether the entire termination of the rectum, including with the diseased part a portion of the adjacent sound bowel, may not be removed." 208.

Mr. Crosse, of Norwich, a very excellent surgeon, as our readers well know, excised a malignant tumour, which grew, like a mushroom, from the left side of the rectum, just within the sphincter. Two months after he left the hospital, this patient returned with great increase of the disease, and he died in five or six months after the operation.

Mr. Mayo inquires whether we may not go farther than this and excise the whole cylinder of the gut, when the disease does not extend very high.

Mr. Mayo has performed this operation on a woman, aged forty. The inner surface of the bowel began to be ulcerated half an inch within the orifice. The ulcer extended round the rectum, and was upwards of an inch in breadth: there was considerable induration. The patient had suffered long and severely, and could not quit the recumbent posture. The patient felt relief after the operation, regained her embonpoint, and neither contraction, nor incontinence of fæces resulted. But a serious evil followed. Prolapsus of the bowel came on; some length of intestine was gradually pushed out in a state of eversion; and the mucous surface, irritated by exposure and pressure, became a new and constant source of uneasiness. Two years after the operation the woman died of peritoneal inflammation. The mucous membrane adjoining the cicatrix had begun anew to ulcerate.

It may be questioned whether the operation was even in this case a benefit. One evil was substituted for another, and the patient died at last, with the disease returning, having undergone the pain and the risk of an operation, and suffered all the feelings of disappointment that an operation uselessly submitted to engenders. When we compare this with the case of Mr. Crosse—when we reflect on the ill success of operations for scirrhus under the most favourable circumstances, for instance, in the testis and mamma—when we consider that a patient is not likely to submit to excision of a portion of the rectum, and indeed that the surgeon is not warranted in proposing it until the disease is unequivocally established—and when we take into the account the unfavourable impression that useless or unsuccessfull operations create, not only against the operator, but against his art—when we look at all these circumstances we must say that we can hardly join Mr. Mayo in his feeling in favour of an operation.

Mr. M. concludes with the narration of a case, and expresses a regret that he did not operate at an earlier period. We have seen a good deal of operations at all periods for malignant disease, and we fear the result would have been the same, had Mr. Mayo's wish been realised.

In this case, when the bowels acted, a peculiar substance came down, in texture not unlike a common polypus of the nose, but it had greater firmness. The disc of the tumour was more vascular than the pedicle, and it bled readily upon being handled. It was quite insensible. Upon examining the rectum, the pedicle of the tumour was found attached to the fore-part of the bowel, behind the prostate gland. There was some little hardness of the rectum at this part. Now this really appears to have been, comparatively speaking, a favourable case. Yet, mark the result. The tumour was removed by a ligature. In three months the patient came back with the fore-part of the rectum occupied by nodular masses, appearing to the touch, of the same substance as the original tumour. Mr. M. included all these in a ligature. The tumour again grew rapidly, and the patient died. The disease was fungoid scirrhus.

The fact of the matter is that a great series of experiments on the extirpation of malignant disease, have been made of late years. Some have selected the jaws, some the malar bone, some the extremities, some the uterus, and some the rectum, for their operations. The issue of the cases is graven on the tomb-stone.

We have now completed our notice of Mr. Mayo's work. In a second edition, which we hope to see soon, we have no doubt the subjects will be considered more systematically, that several omissions, which we observe, will be supplied, and that the wood-cuts, which have rather a ridiculous appearance, will be omitted. We put it to Mr. Mayo's good taste to decide, whether any possible advantage can result from giving a sketch of a pile with a needle through it, or from a scratchy representation of what is denominated "a forest of warts." We trust that when Mr. Mayo has seen more of these diseases he will gratify the profession by laying before it the results of his extended experience.

